# Supplementary material for: Generating Bone Marrow Chimeric Mouse Using GPR120 Deficient Mouse for the Study of DHA Inhibitory Effect on Osteoclast Formation and Bone Resorption
Source: Int J Mol Sci. 2023 Nov 30;24(23):17000. doi: 10.3390/ijms242317000 (PMC10707107; doi:10.3390/ijms242317000)
Supplement: Supplementary file 1 [file ijms-24-17000-s001.zip › ijms-2648776-supplementary.pdf]

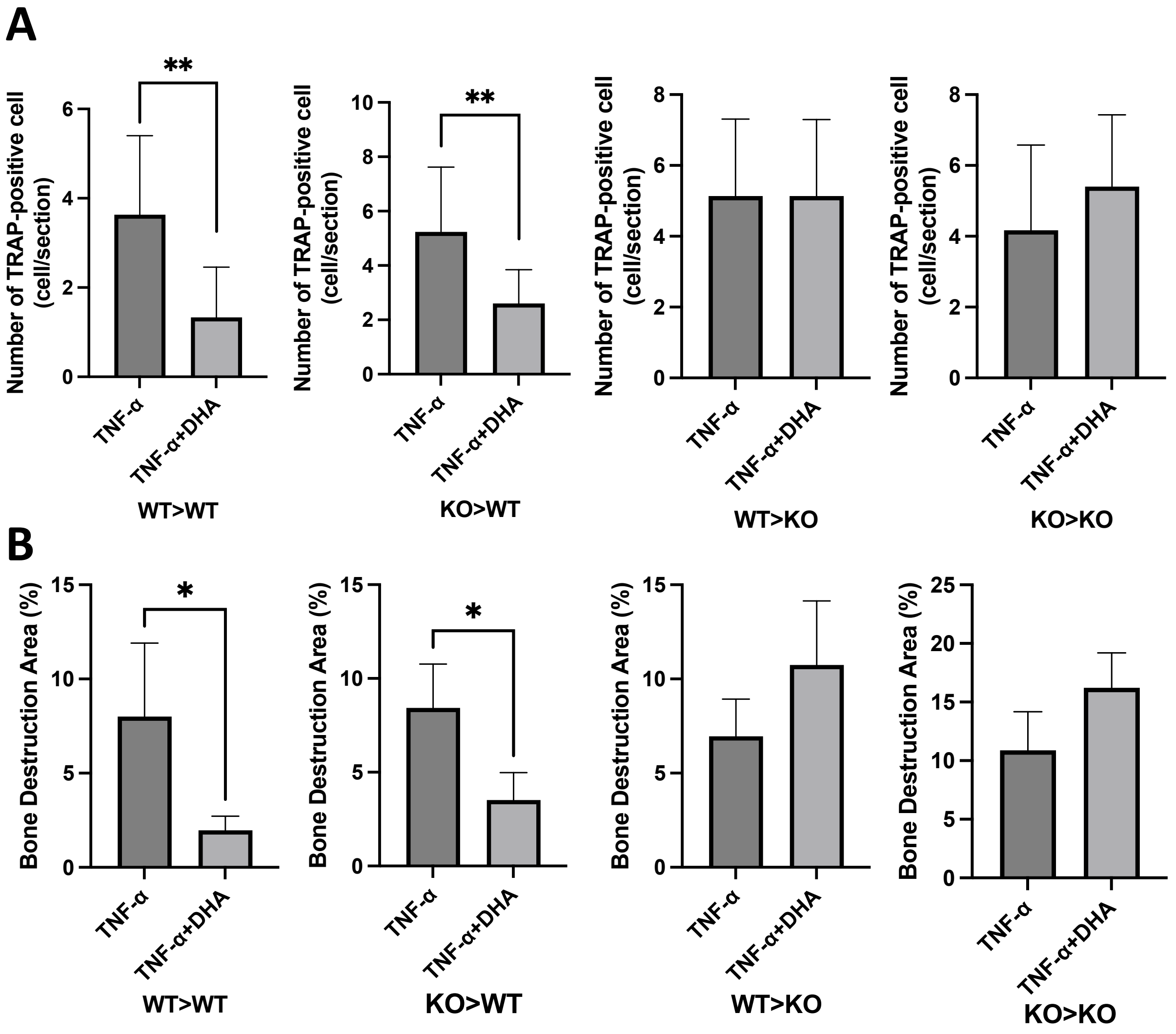

Figure S1. Comparison between TNF-α single administration and TNF-α and DHA co-administration groups, in (A) the number of TRAP-positive cells and (B) the percentage of bone resorption in the sagittal suture. DHA significantly decreases the osteoclast formation and bone resorption in WT>WT and KO>WT groups but not in WT>KO or KO>KO groups. The bars are expressed as means and the error bars represent SD. The statistical significance of differences was determined with T tests. (* $P < 0.05$, ** $P < 0.01$; $n = 4$).

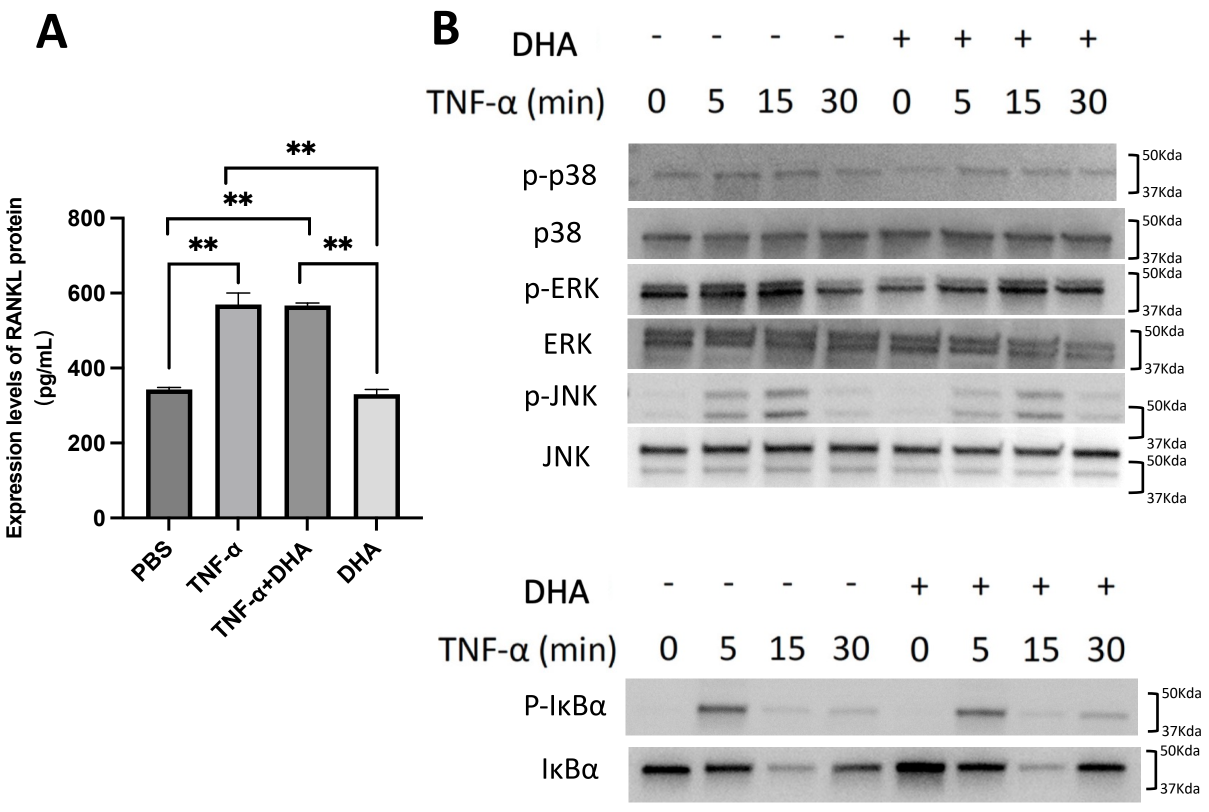

Figure S2. DHA does not suppress TNF-α triggered osteoblast related RANKL expression in GPR120-KO osteoblasts. DHA did not exert obvious effects on TNF-α-induced MAPKs and IκB activation in GPR120-KO osteoblasts. (A) The expression level of RANKL protein inGPR120-KO osteoblast was detected by ELISA. (B) Osteoblasts were incubated with TNF-α (100 ng/mL) with DHA (100 ng/mL) or TNF-α (100 ng/mL) only. Osteoblasts were lysed and then analyzed by Western blotting using p38, phospho-p38, ERK1/2, phospho-ERK1/2, JNK, phospho-JNK ;IkB and phospho-IkB antibodies. The bars are expressed as means and error bars represent SD. The statistical significance of differences was determined with Scheffé's tests. (* $P < 0.05$, ** $P < 0.01$; $n = 4$).
